# Oxidative Stress and Ultrastructural Changes in Laminar Tissue of Dairy Cows with Acute Laminitis Induced by Oligofructose Overload

**DOI:** 10.3390/ani16060980

**Published:** 2026-03-20

**Authors:** Muhammad Abid Hayat, Jiafeng Ding, Xianhao Zhang, Tao Liu, Jiantao Zhang, Hongbin Wang

**Affiliations:** 1 Department of Veterinary Surgery, College of Veterinary Medicine, Northeast Agricultural University, Harbin 150030, China; abid.hayat@uvas.edu.pk (M.A.H.); zhxh950717@outlook.com (X.Z.); liutaotiger@163.com (T.L.); zhangjiantao@neau.edu.cn (J.Z.); 2 Heilongjiang Key Laboratory for Laboratory Animals and Comparative Medicine, Harbin 150030, China; 3 Jiangsu Key Laboratory of Medical Science and Laboratory Medicine, School of Medicine, Jiangsu University, Zhenjiang 212013, China; 4 College of Animal Science and Technology, Guangxi University, Nanning 530004, China; jiafengding@cau.edu.cn

**Keywords:** acute laminitis, dairy cows, laminar tissue, oligofructose, oxidative stress, hemidesmosomes

## Abstract

This study explored ultrastructural changes and the expression of oxidative stress-related genes and proteins in the laminar tissue of dairy cows with acute laminitis induced by oligofructose (OF) overload. Twelve clinically healthy, non-pregnant Chinese Holstein cows were randomly allocated into two groups: the OF-overload group (*n* = 6) and the control group (*n* = 6). 17 g/kg BW of oligofructose (OF) dissolved in 20 mL/kg BW of deionized water was provided to the OF-treated group, while the control group received 20 mL/kg BW of deionized water via a stomach tube. Laminar tissue samples were collected at 72 h post-OF administration. RT-qPCR revealed significantly increased *Keap1* mRNA expression (*p* = 0.0097) and significantly decreased *Nrf2* (*p* < 0.0001), *Ho1* (*p* < 0.0001), and *Nqo1* (*p* = 0.0101) mRNA expression in the OF group compared to the control group. Western blot analysis confirmed corresponding protein-level changes, with significantly increased *Keap1* (*p* = 0.0062) and significantly decreased *Nrf2* (*p* = 0.0008), *Ho1* (*p* = 0.0297), and *Nqo1* (*p* = 0.0004) in the OF group compared with the control group. Immunohistochemical analysis revealed significantly increased cytoplasmic *Keap1* distribution (*p* = 0.0200) and significantly decreased nuclear *Nrf2* localization (*p* = 0.0032) in the OF group than the control group. Ultrastructural examination revealed significant pathological changes in the OF group, including a reduced number of hemidesmosomes (*p* < 0.01), an increased distance from epidermal basal cells to the lamina densa (*p* < 0.01), thickened and damaged lamina densa with disorganized collagen fibers, and deformed basal cell nuclei with reduced chromatin relative to the control group. In conclusion, these findings demonstrate that OF-induced acute laminitis is associated with significant dysregulation of the *Keap1-Nrf2* antioxidant pathway and severe ultrastructural damage to the dermal–epidermal interface, suggesting that oxidative stress contributes to laminar tissue injury in dairy cows.

## Simple Summary

Bovine laminitis is the leading cause of lameness, resulting in significant financial losses and animal welfare issues in the worldwide dairy sector. Indeed, its pathogenesis remains poorly understood. Oxidative stress may play an important role in the pathophysiology of dairy cow laminitis. This study evaluated the oxidative stress status in the laminar tissue of dairy cows with oligofructose (OF)-induced laminitis at the gene and protein levels. Decreased gene and protein expression levels of nuclear factor erythroid 2-related factor 2 (*Nrf2*), heme oxygenase-1 (*Ho1*), and NAD(P)H: quinone oxidoreductase 1 (*Nqo1*) were observed in the OF group’s laminar tissue of dairy cows. However, the gene and protein expression levels of Kelch-like ECH-Associated protein 1 (*Keap1*) were enhanced. The distribution of *Keap1* expression increased, while that of the *Nrf2* decreased in the OF group relative to the control group. In addition, ultrastructural analysis of the laminar tissue showed that the OF led to shrinkage, loss of hemidesmosomes (HDs), and rupture of anchoring fibers. These findings suggest that the unbalanced expression of oxidative stress-associated genes and proteins may contribute to epidermal detachment, confirming that oxidative stress was enhanced in the laminar tissue of OF-treated cows. Therefore, targeting the *Keap1-Nrf2* pathway may lead to improved prevention and treatment strategies for bovine laminitis. However, further large-scale gene- and protein-based research is needed to fully understand the pathophysiology of dairy cows’ laminitis.

## 1. Introduction

Laminitis is a hoof disease that causes lameness [1]. It was first described as diffuse aseptic pododermatitis, characterized by inflammation of the dermal layers within the hoof [2]. The cause of laminitis is multifactorial, with excessive intake of rapidly digestible carbohydrates as one of the major contributing factors [3]. There have been significant economic losses caused by laminitis, which may dramatically limit the growth of dairy farming. In the past decade, many studies have focused on the pathophysiology of milk cow laminitis [4,5], metabolomic [6], histology [7], and proteomics [8]. However, due to differences in clinical investigations and a lack of valid laboratory models [9], the root cause and pathophysiology of acute bovine laminitis remain unknown. In clinical practice, bovine laminitis is classified as a secondary cause of several inflammatory illnesses, including ruminal acidosis, mastitis, metritis, and Gram-negative pleuropneumonia [10]. By simulating such clinical conditions, investigational models of dairy cow laminitis have been formulated. The OF-induced model is more precise and more widely applied than other models of inducement [11,12], and has been confirmed to exhibit identical clinical presentations and typical histopathological changes to the acute laminitis cases [13,14]. Although the model developed by OF has been more widely applied in equine laminitis studies [15,16], its use in bovine laminitis has allowed a systematic exploration of the molecular pathogenesis of this disease.

The researchers have discovered evidence that dairy cow laminitis influences the structural integrity of the lamellae. These tissues are pertinent to offer proper orientation of the third phalanx (P3) and transmit major mechanical forces to the hoof when they attach P3 to the hoof capsule [17]. The lamellae structure is organized into epidermal lamellae and dermal lamellae, linked by a contact zone called the basement membrane (BM) [5]. When epidermal and dermal lamellae are disrupted, P3 sinks and turns within the capsule, resulting in severe lameness and pain [18]. The extracellular matrix (ECM) is a major component of the BM, which serves as a boundary between two different layers. The two principal histological alterations that are considered to result in epidermal detachment in bovine laminitis are BM destruction and separation [19,20]. Nevertheless, the underlying molecular mechanisms driving these structural changes remain unclear.

Oxidative stress, defined as an imbalance between reactive oxygen species (ROS) production and antioxidant capacity, has emerged as a potential contributor to laminitis pathophysiology. Increased ROS under oxidative stress causes damage to cellular macromolecules, leading to protein alteration, lipid peroxidation, and DNA damage [21,22], resulting in hoof tissue dyskeratosis [23] and programmed chondrocyte death [24]. Current research suggests that oxidative stress is closely related to bovine laminitis [25,26,27]. In the face of deleterious signals, the body had an adjusted oxidant scavenging system. The role of this step has been demonstrated by several recent studies, and both *Keap1* and *Nrf2* are important proteins in cellular defense against oxidative stress. Second-phase enzymatic systems, characterized by the presence of *Nqo1* and *Ho1*, exert antioxidant, and anti-inflammatory activity in conditions predisposing to oxidative stress [28]. This is mediated by *Nrf2* binding to an antioxidant response element (ARE) [29]. Downstream genes of *Nrf2*, such as *Ho1* and *Nqo1*, also have antioxidant activity [30]. *Keap1* is a cytoplasmic chaperone protein and secondary structure *Nrf2*-regulating domain that is specifically related to the inhibition of *Nrf2* by receptors. *Nrf2* is negatively regulated by *Keap1* [31]. Nrf2 is sequestered in the cytoplasm under basal conditions and attaches to the actin cytoskeleton via *Keap1*, facilitating its proteasomal degradation [29]. Structural changes in the *Keap1* upon oxidative stimulation release *Nrf2*, which translocates to the nucleus. *Nrf2* binds to antioxidant response elements (ARE) in the promoter of target genes in the nucleus, initiating the transcription of phase II-detoxifying enzymes and antioxidant proteins [29]. The major downstream targets are heme oxygenase-1 (*Ho1*) and NAD(P)H: quinone oxidoreductase 1 (*Nqo1*), which have significant cytoprotective functions [31]. The *Keap1-Nrf2* signaling pathway thus plays a critical role in maintaining cellular redox homeostasis and protecting against oxidative stress-induced tissue damage [32].

Although oxidative stress has been well-characterized in equine laminitis [33,34], the molecular mechanism underlying oxidative damage in bovine laminar tissue remain undefined. To date, no research has comprehensively investigated the *Keap1-Nrf2* antioxidant pathway and its downstream effectors in the laminar tissue of dairy cows with acute laminitis, along with ultrastructural alterations in the dermo–epidermal interface. Therefore, this study examined the oxidative stress response for the first time at both gene and protein levels as well as ultrastructural alterations in the laminar tissue of dairy cows with acute laminitis induced by OF overload. By integrating molecular and structural analyses, this study aims to characterize laminitis pathophysiology and identify potential therapeutic targets for this economically devastating condition. We hypothesized that oral OF challenge induces oxidative stress and ultrastructural alterations in the laminar tissue of bovine claws through dysregulation of the *Keap1-Nrf2* pathway.

## 2. Materials and Methods

### 2.1. Experimental Animals

Twelve clinically sound non-pregnant Chinese Holstein cows, which have normal locomotion [35], and have not received a history of claw horn lesions, were used in this experiment. The cows’ body weight ranged from 335 to 403 kg (379.71 ± 19.87 kg), age ranged from 18 to 26 months (20.67 ± 3.01 mo), and BCS [36] ranged from 2.7 to 3.3 (3.00 ± 0.23). All the cows selected for this experiment were purchased from the Qingxi dairy farm in Xiangfang District, Harbin, China. The study’s cows were placed in the animal shelter with a rubber floor for thirty days prior to the trial. The cows had unlimited access to assorted forages ad libitum and a sufficient supply of clean drinking water. The experimental cows were recorded daily for their hoof temperature, body temperature, and blood pressure, as well as walking for 5 min, to assess their health.

### 2.2. Experimental Design and Treatment

Twelve dairy cows were randomly allocated into two groups: the OF-treated group (*n* = 6) and the control group (*n* = 6). 17 g/kg BW of oligofructose (Shandong Shenglong Technology Co., Ltd., Jinan, China) dissolved in 20 mL/kg BW of deionized water was administered to the OF-treated group, and 20 mL/kg BW of deionized water was administered to the control group at 0 h using a stomach tube (length 2.2 m, diameter 25 mm) in compliance with the protocol reported by [11,12]. Oligofructose (5%) was provided orally once daily for 3 days. The cows were trained for the clinical examination before receiving the advanced oral dose of OF.

Claudication assessments were performed at −72, 0, 6, 12, 18, 24, 36, 48, 60, and 72 h. During this time, cows were trained to walk and trot along a straight path by hand before turning in a small circle on the same ground at the Animal Hospital at Northeast Agricultural University in Harbin, China. Five certified veterinarians rated each cow’s claudication scores using the protocol described by Sprecher et al. [35]. Once all certified vets scored a score of ≥2, the cow was classified as lame. On each 6 h, all cows were recommended to the clinical evaluation, including respiratory rate, heart frequency range, rectal temperature, eating routine, feces consistency, hoof coronary band, weight shift, diastolic blood pressure, hoof temperature, rumen movements, hoof discomfort, and rumen pH. After 72 h of OF-overloading, each cow was euthanized by overdosing (20 mg/kg) with pentobarbital sodium and phenytoin sodium (Fatal-plus; 20 mg/kg IV; Vortech Pharmaceuticals, Ltd., Dearborn, MI, USA) by intravenous injection [13], and confirmatory methods (pupillary reflex, auscultation) were performed by following the Institutional Animal Care and Use Committee, and AVMA and OIE guidelines for humane euthanasia. In accordance with cow welfare guidelines, supportive therapy with Ringer lactate (15 mL/kg of BW; Heping Animal Medicine Co., Ltd., Harbin, China) at 18 and 24 h (maintained hydration and electrolyte balance) and calcium borate (14 mg of Ga/mL; 1.4 mL/kg of BW; Heping Animal Medicine Co., Ltd., Harbin, China) at 18 h (prevented hypocalcemia during metabolic stress) was provided post-OF administration.

### 2.3. Laminar Tissue Sampling

In accordance with the protocol presented by Thoefner et al. [13], the deceased animals’ left hind claws were separated in 5–10 min, positioned in an iced pack, and immediately transported to the laboratory. Laminar tissue was exposed by cutting the hoof wall, and the hoof capsule was detached. Subsequently, a laminar tissue was cut into small tissue sections (1–2 mm^2^). It was immediately frozen in a liquid nitrogen and stored at −80˚C. The whole process was carried out in ice. To avoid tissue exposure, proper gloves and sterile masks were used. Hoof capsule contains the laminar wall tissue which is closely attached to it. The lamella wall typically encompasses the whole of the axial, abaxial, and dorsal side of the claw, extending between 3 and 7 cm between the coronet and the sole corium. The dissociated lamella wall which is excised 2 cm below the coronet encompasses the axial and abaxial elements of the laminar wall tissue. The ideal laminar wall tissue dimension is of length (6 × 3 cm) and width (3 cm).

### 2.4. Transmission Electron Microscopic Structure Observation of Laminar Tissue of Dairy Cows

The ultrastructure of hoof tissue in dairy cows with laminitis was observed using a transmission electron microscope. The precise operations steps were as follows: The small pieces of hoof tissue were removed, fixed in 2.5% glutaraldehyde solution, refixed with 1% osmium tetroxide solution at 4 °C for 2 h, dehydrated with graded acetone concentrations, and then embedded in 812 epoxy resin (Polysciences, Inc., Warrington, PA, USA). At 70 °C, the tissue block was polymerized in resin for 2 days, and the tissue was cut into 65–70 nm thick slices using a UC6 ultramicrotome (Leica Microsystems, Vienna, Austria) and stained with uranyl acetate and lead citrate. An H-7650 electron microscope (Hitachi High-Tech Corporation, Tokyo, Japan) was then used to observe and measure the ultrastructure of hemidesmosomes, organelles, and nuclei of the basal cells of the epidermis of the hoof leaf, as well as the distance from the basal cells to the compact layer of the hoof leaf. The basement membrane hemidesmosomes counting method was performed as follows: at approximately 30,000 magnifications, 100 μm of the hoof tissue basement membrane of each cow in the both groups was continuously photographed, the hemidesmosomes on the basement membrane were observed, and the number of hemidesmosomes per micron was calculated. The distance from the basal cells to the dense layer of the hooves was measured as follows: A measurement point every 1 μm was placed on the 100 μm long basement membrane to measure the distance perpendicular to the direction of the basal cell membrane from the cell membrane to the central area of the hoof dense layer.

### 2.5. RNA Isolation and cDNA Synthesis

The total RNA was isolated from laminar tissue of 12 cows using the RNA Miniprep-Kit (Invitrogen, Carlsbad, CA, USA) according to the manufacturer’s instructions. The tissue samples (100 mg) were mixed with 1 mL TRIzol reagent (Invitrogen, Carlsbad, CA, USA). Then it was put into a non-DNAse/RNAse centrifuge, and the total RNA without DNA, protein, and isopropanol precipitates were removed using chloroform and washed with 75% ethanol. The quality and quantity of separated total RNA were assessed with an ultra-nucleic acid protein testing kit (Thermo Fisher Scientific, Waltham, MA, USA). The accuracy of each RNA sample was checked by 1% agarose gel electrophoresis (Bio-Rad Laboratories, Hercules, CA, USA). RNA samples were diluted to 1 μg/μL using optical density measurements. 1 μg total RNA was isolated from each sample according to protocols established by the manufacturer of the Prime-Script™ RT Kit (Takara, Dalian, China). Complementary DNA (cDNA) was obtained using reverse transcription. During Real-time RT-qPCR analysis, the resulting cDNA was diluted (1:3) with DEPC water and kept at −20 °C till further utilization. DEPC water (Beijing Bio-Top Technology Co., Ltd., Beijing, China) was prepared by mixing dH_2_O and DEPC, then sterilized to remove DEPC.

### 2.6. Quantitative Real-Time Polymerase Chain Reaction (RT-qPCR)

In this experiment, primers were prepared to identify the genes *Keap1*, *Nrf2*, *Ho1*, *Nqo1*, and *GAPDH*, which were designed by Shanghai Sheng Gong Biotechnology, Co., Ltd. (BBI Life Sciences, Shanghai, China) (Table 1). The efficiency of each primer sequence was assessed using the Blast Computer Program of the NCBI (National Center of Biotechnology Information) database (Bethesda, MD, USA).

The RT-qPCR experiment was carried out with the Green chimeric fluorescence detection procedure of SYBR Premix Ex Taq^TM^‖Kit (Takara, Dalian, China) by the Light Cycler 480 RT-qPCR system (Roche, Mannheim, Germany). The PCR reaction mixture (20 μL final volume) contains 2 μL cDNA template and 18 µL of the major blend to be used in the PCR. Subsequently, the PCR master cocktail was composed of 6.4 μL of DEPC water, 10 μL of SYBR green fluorescent agent, and 1.6 μL of general primer solution (10 μM each of forward and reverse primers). The end primer concentration was 0.4 μM/μL. The PCR protocols are as follows: pre-denaturation, 1 cycle, 95 °C, 1 min; quantitative estimation, 40 cycles of 95 °C, 5 s, and 60 °C, 1 min; melting curve analysis, 95 °C, 5 s, 60 °C, 1 min, 95 °C, 1 cycle; cooling, 1 cycle at 50 °C for 30 s. Ct values of the different genes were calculated using LightCycler 480 software 2.0 (Roche, Mannheim, Germany) and the Abs Quant/Fit points method. PCR efficiency of the individual genes was calculated using the △△Ct technique with *GAPDH* as the internal reference gene.

### 2.7. Western Blot

The preserved laminar tissue (100 mg) was added to RIPA lysis buffer (Beyond Biotech, Shanghai, China). The primary constituents of RIPA buffer were deoxycholate (1%), Triton X-100 (1%), and sodium lauryl sulfate (0.1%). Following the administration of 10 μL of phenylmethylsulfonyl fluoride (protease inhibitor, Beyond Biotech, Shanghai, China), the tissue was processed in a grinder (4 °C, 4 min), then spun (12,000 rcf/min, 15 min). The protein concentration was assessed using the BCA method (Beyotime Biotechnology, Shanghai, China). The protein volume was determined in the tissue sample using SDS-PAGE where 10 μL of the protein sample was added to each well. The overall protein concentration was estimated at 25 μg. The target proteins were transferred to nitrocellulose (NC) filter membranes (Pall Life Sciences Inc., Pensacola, FL, USA) after isolation on 10% polyacrylamide gel following the semi-dry method (300 mA, 1.5 h). To block the membrane, it was gently agitated in 5% nonfat milk in TBS (Tris-buffer saline; Leagene Biotechnology, Beijing, China) with 0.1% Tween-20 (0.1% Tween-20 TBS, TBST; Leagene Biotechnology, Beijing, China) for 2 h at room temperature. The blocked membranes were then subjected to primary antibodies comprising *Nrf2* (1:750), *Keap1* (1:500), *Ho1* (1:500), *Nqo1* (1:750), and a 1:2000 dilution of *β*-actin (BIOSS Antibodies Beijing Biosynthesis Biotechnology Co., Ltd., Beijing, China), then incubated at 4 °C for 12 h and washed three times with TBST (15 min per wash). It was then combined with secondary antibody (HRP-labeled goat anti-rabbit IgG) at a ratio of 1:10,000 (in 1 X TBST, BBL Life Sciences, Shanghai, China) and kept at room temperature (2 h) followed by shaking (2 h, room temperature). The NC membrane was also rinsed three times with TBST (15 min each) through rocking. Then, it was put on the plate of a TANON 5200 exposure apparatus (Shanghai Tanon Technology Co., Ltd., Shanghai, China), and filter paper was used to remove the liquid. The ECL liquid (Meilun Biotechnology Co., Ltd., Dalian, China) was then dropped to overlay the protein bands uniformly. An image of the Western blot was taken with its corresponding exposure time. ImageJ software version 1.53t. was used to determine the gray value of each protein in each gel, and the gray ratio of the target protein band to the internal reference protein band was calculated to assess the relative expression of the target protein.

### 2.8. Immunohistochemistry

Immunohistochemistry was utilized to assess the expression of *Keap1* and *Nrf2* in laminar tissue. The samples were cut into the proper sizes, fixed (4% paraformaldehyde, 24 h), and then sliced and implanted. The tissue sections were paraffinized by overnight incubation at 80 °C, followed by a 10 min incubation in 3% H_2_O_2_ in the dark to inactivate endogenous peroxidase activity, and antigen retrieval was performed in a pressure cooker with sodium citrate buffer. The resulting fragments were incubated with bovine serum albumin (20 min, room temperature), primed overnight (4 °C) with a primary antibody mixture (1: 200) dilution against *Keap1* and *Nrf2* (Novus Biologicals, Littleton, CO, USA), and then incubated with streptavidin-conjugated horseradish peroxidase (30 min, room temperature). The tissue sections were subsequently glued with neutral glue, stained with hematoxylin and DAB, and stored in an oven. Finally, the stained tissue segment in each group was assessed under a microscope and analyzed using Image-Pro Plus version 6.0 software-IPWIN16.EXE (Media Cybernetics, Rockville, MD, USA).

### 2.9. Statistical Analysis

The data analysis was carried out using GraphPad Prism (Version 8.01, GraphPad software, Inc., San Diego, CA, USA). Normality of data distribution was assessed using the Shapiro–Wilk test, which confirmed that all variables followed a Gaussian distribution. The 2^−ΔΔCt^ method was used to calculate changes in relative gene expression. To evaluate local histology results, including gene expression, protein expression, immunohistochemical staining, hemidesmosome counts, and basement membrane distance, an independent Student’s *t*-test was used to assess differences between the two groups. For clinical data including longitudinal lameness scores assessed at multiple time points, a two-way repeated-measures ANOVA was performed, followed by Bonferroni’s multiple-comparison test across both groups. The sample size (*n* = 6 per group) was determined based on previous studies using the oligofructose-induced laminitis model [11,12,37]. Differences were considered when *p* < 0.05. All data were indicated as mean ± SD.

## 3. Results

### 3.1. Clinical Manifestation of Dairy Cows Laminitis

All dairy cows treated with OF had clinical manifestations of unique acute ruminal and systemic acidosis like persistent profuse diarrhea, in-appetence, absent dietary intake, depression, anorexia, swelled of carpal (tarsal) joints, inflamed hoof coronary band, disturbed weight shift, elevation of heart rate, elevated diastolic blood pressure, high hoof temperature, elevated body temperature, slow respiration, apathy of digital (toe) arteries, hoof pain, reduced rumen pH, intermittent fever, and lameness [25,27,37]. There were no signs of a systemic illness in the control cows. To examine claudication, clinical indications of laminitis were firstly assessed at 24 h following OF overload and continued to change until the maximum claudication score of 3–5 at 60 h to 72 h [38] was found, which confirms the acute laminitis. These symptoms were all similar to those reported by [11,12,13].

### 3.2. Oxidative Stress-Associated Genes Expression in Laminar Tissue of Laminitis Dairy Cows

**Figure 1:**
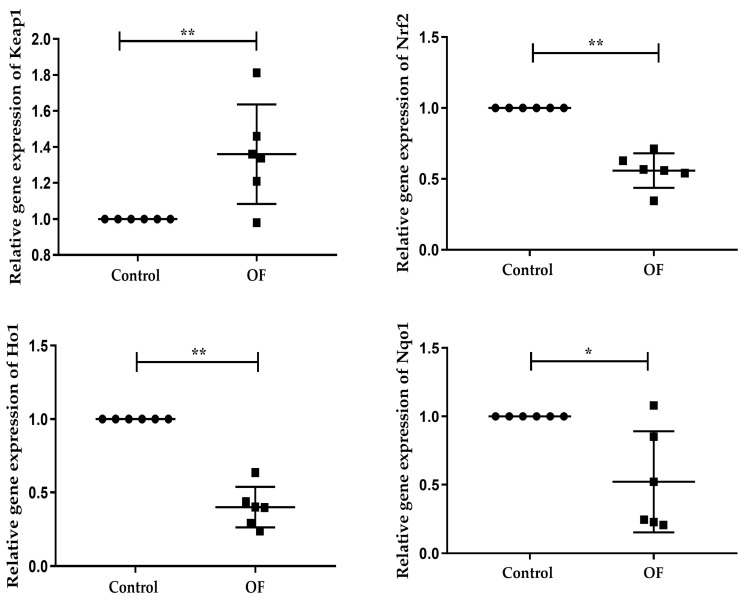
RT-qPCR results of mRNA concentration of oxidative stress-related genes including, *Keap1*, *Nrf2*, *Ho1*, and *Nqo1* in laminar tissue of the both groups. Data were analyzed using Student’s *t*-test and are presented as mean ± SD. “*” show (*p* < 0.05); “**” show (*p* < 0.01).

The expressions levels of oxidative stress-associated genes were assessed by RT-qPCR. The results showed that *Keap1* mRNA expression was significantly increased in the OF group (1.360 ± 0.2765) compared to the control group (1.000 ± 0.000) (*p* = 0.0097). In contrast, *Nrf2* mRNA expression was significantly decreased in the OF group (0.5587 ± 0.1216) relative to the control group (1.000 ± 0.000) (*p* < 0.0001). Similarly, *Ho1* mRNA expression was significantly lower in the OF group (0.3997 ± 0.1381) compared to the control group (1.000 ± 0.000) (*p* < 0.0001), and *Nqo1* mRNA expression was also significantly decreased in the OF group (0.5218 ± 0.3705) than the control group (1.000 ± 0.000) (*p* = 0.0101) (Figure 1). These results indicate that oral OF treatment induces oxidative stress in the laminar tissue of laminitis dairy cows.

### 3.3. Oxidative Stress-Associated Proteins Expression in Laminar Tissue of Laminitis Dairy Cows

**Figure 2:**
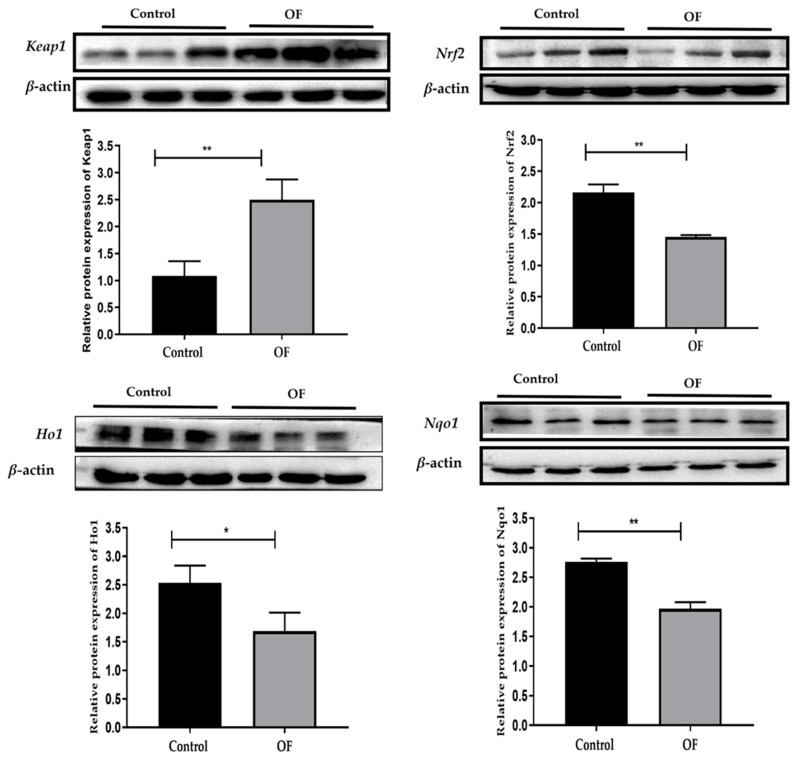
Western blot results of oxidative stress-related proteins expression including *Keap1*, *Nrf2*, *Ho1*, and *Nqo1*, in the laminar tissue of both groups. Data were analyzed using Student’s *t*-test and are presented as mean ± SD. “*” show (*p* < 0.05); “**” show (*p* < 0.01).

Western blot examination revealed that *Keap1* protein expression was significantly increased in the OF group (2.498 ± 0.3770) compared to the control group (1.087 ± 0.2700) (*p* = 0.0062). Conversely, *Nrf2* protein expression was significantly decreased in the OF group (1.455 ± 0.02786) relative to the control group (2.162 ± 0.1296) (*p* = 0.0008). *Ho1* protein expression was significantly lower in the OF group (1.687 ± 0.3238) compared to the control group (2.533 ± 0.3023) (*p* = 0.0297), and *Nqo1* protein expression was also significantly decreased in the OF group (1.966 ± 0.1108) than the control group (2.761 ± 0.05577) (*p* = 0.0004) (Figure 2). These findings demonstrate that oral OF treatment induced oxidative stress in laminar tissue at the protein level, consistent with gene expression results that suggested the involvement of ROS-mediated inflammation as an important pathway leading to laminitis. The detailed western lot pictorial representation for *Keap1*, *Nrf2*, *Nqo1*, and *Ho1* is provided in the Supplementary Material (Figures S1–S4).

### 3.4. Immuno-Expression of Keap1 and Nrf2 Proteins in Laminar Tissue of Laminitis Dairy Cows

**Figure 3:**
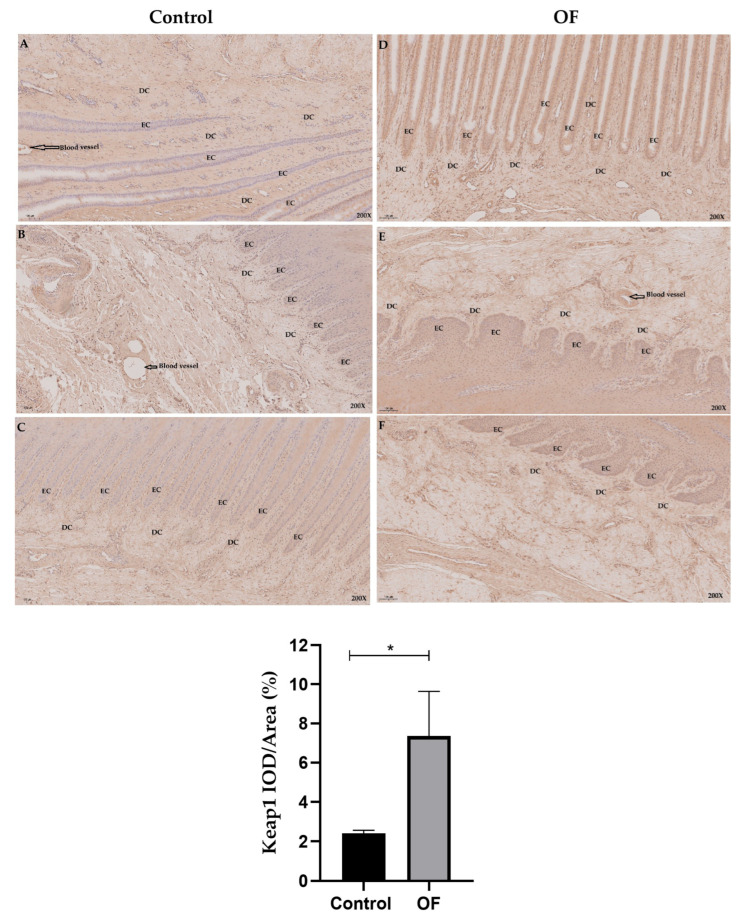
Immunohistochemical staining of *Keap1* in laminar tissues: Scale = 100 μm, 200×, (**A**–**C**) control cows; (**D**–**F**) OF-treated cows. EC, epidermal cels of epidermal lamellae; DC, dermal cells of dermal lamellae. Data were analyzed using Student’s *t*-test and are presented as mean ± SD. “*” show (*p* < 0.05).

The immunohistochemical analysis of *Keap1* protein expression in cow laminar layers revealed that mean *Keap1* staining within the cytoplasm of laminar tissue in the OF-treated group measured approximately 7.36%, while staining measured around 2.41% in control tissue. Statistically, *Keap1* protein expression in the cytoplasm of laminar tissue was significantly higher in the OF-treated group (7.356 ± 2.284%) compared to the control group (2.405 ± 0.1598%) (*p* = 0.0200), as indicated in Figure 3. The increased cytoplasmic *Keap1* distribution suggests enhanced sequestration of *Nrf2.*

**Figure 4:**
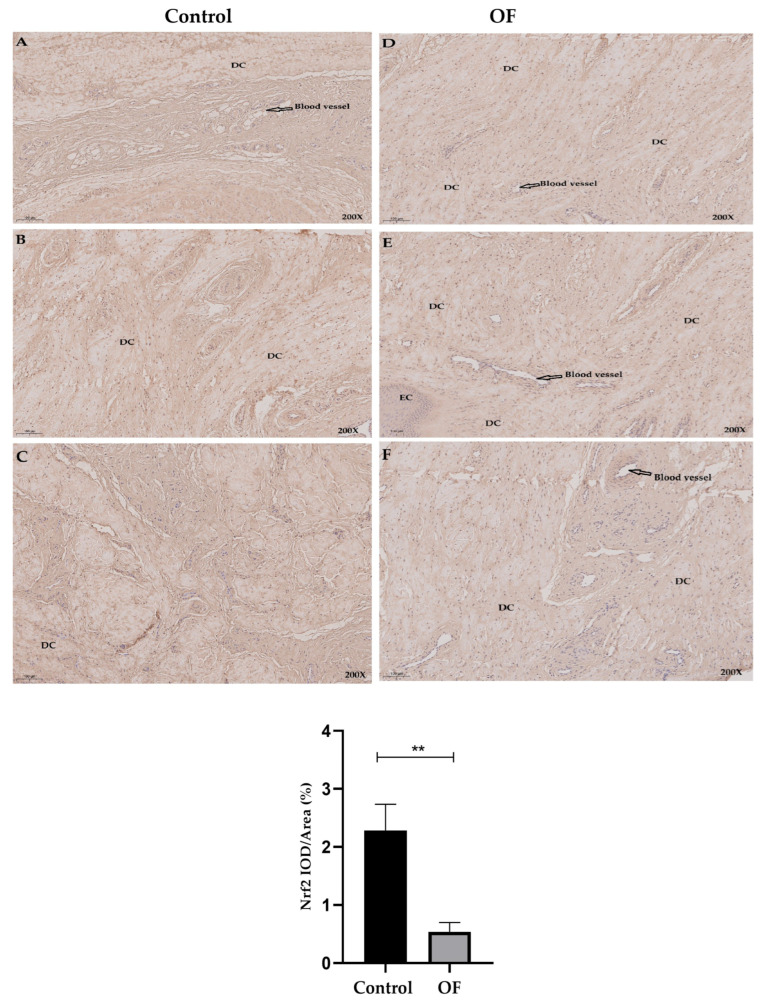
Immunohistochemical staining of *Nrf2* in laminar tissues: Scale = 100 μm, 200×, (**A**–**C**) control cows; (**D**–**F**) OF-treated cows. DC, dermal cells of dermal lamellae. Data were analyzed using Student’s *t*-test and are presented as mean ± SD. “**” show (*p* < 0.01).

The immunohistochemical findings of *Nrf2* protein expression showed that the average *Nrf2* staining in the nucleus of laminar tissue in the OF group was 0.53%, while it was 2.29% in the control tissue. Statistically, *Nrf2* protein expression in the nucleus was significantly lower in the OF-treated group (0.5375 ± 0.1610%) compared to the control group (2.286 ± 0.4498%) (*p* = 0.0032), as indicated in Figure 4. This marked reduction in nuclear *Nrf2* localization indicates impaired activation of this transcriptional factor despite the presence of oxidative stress, further confirming that oxidative stress is involved in laminar tissue damage during acute laminitis.

### 3.5. The Ultramicroscopic Structure Characteristics of Laminar Tissue of Laminitis Dairy Cows

**Figure 5:**
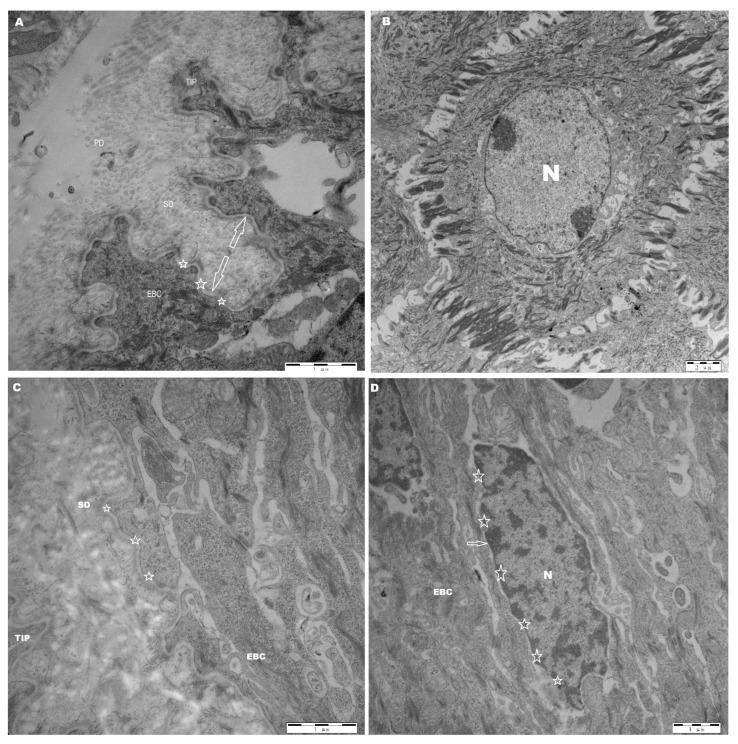
Ultramicroscopic characteristics of laminar tissue of control and OF-treated cows. (**A**,**B**): control group, (**C**,**D**): OF-treated group. EBC: epidermal basal cell; N: nucleus; PD: primary dermal; SD: secondary dermal; Tip: the top of the epidermal basal cell; hemidesmosomes (☆); the separation between lamina densa and epidermal basal cell (arrowhead).

In the control group, the epidermal basal cell’s apex in the laminar tissue were blunt. The dense layer of the leaf was closely connected, deeply stained, and completely linear, running parallel to the basal cell membrane of the epidermis. Many hemidesmosomes were evenly distributed (3.831 ± 1.189) along the basal cell membrane and a closely attached lamina densa (0.068 ± 0.022). The nuclei of epidermal basal cells are oval in shape, located away from the basement membrane, with clear borders and high chromatin density (Figure 5A,B) (Table 2).

In the OF-treated cows, the tip of the basal cells of the epidermis in the laminar tissue was sharpened. The lamina densa appears thick and damaged, with interwoven collagen fibers and is lightly stained. The quantity of hemidesmosomes on the epidermis’ basal cell membrane declined (1.53 ± 1.066) (*p* < 0.01), and their distribution was uneven. The distance between the epidermal basal cell and the lamina densa has risen (0.098 ± 0.029) (*p* < 0.01) dramatically (Table 2). There are a few cytoskeleton anchoring filaments with damaged basal cell organelles. Similarly, in dairy cows with laminitis, the nuclei of epidermal basal cells are deformed, unusually close to the basement membrane, with ambiguous borders and reduced chromatin (Figure 5C,D).

## 4. Discussion

Acute bovine laminitis, which contributes significantly to the gradual degradation of hoof function and structure, is a constant characteristic of oxidative stress. According to published scientific papers, we observed that dairy cow laminitis model was effectively developed through the OF-overload method, and OF group cows indicated clinical features of distinctive acute ruminal and systemic acidosis symptoms, as well as apparent histological indications in the lamellae; for example, BM damage and separation, sinking of the epidermal lamellae, along with changes in basal cell shape [25,37]. The current research extends these findings by investigating the ultrastructural nature of change and oxidative stress-related indicators at the gene and protein levels in laminar tissue during OF-induced dairy cow laminitis, which surpasses our earlier published article that reported the elevated levels of malondialdehyde (MDA) and reduced levels of superoxide dismutase (SOD), catalase (CAT), and glutathione (GSH) in the same experimental model [25].

Research suggests that oxidative stress is closely related to bovine laminitis [25,26,27]. To clarify the effect of oxidative stress and its persistent associated inflammation, ROS induces a compensatory response by inducing *Nrf2* and subsequent expression of antioxidant and detoxifying enzymes [39,40]. Indeed, some *Nrf2*-activating phytochemical anti-inflammatory and antioxidant agents can boost cellular resistance to oxidative and electrophilic insults [41]. Oxidative damage and downregulation of the ROS-producing enzyme *Nqo1* in the laminar tissue were demonstrated in OF-treated cows studied 72 h after OF overload. There has been comprehensive research into the interaction of *Keap1-Nrf2* pathways with multiple refractory diseases, including lung cancer, pulmonary fibrosis, bronchial asthma, and chronic obstructive pulmonary disease (COPD) [42]. The effects of laminitis on the *Keap1-Nrf2* system and its downstream gene products are unidentified and have been studied for the first time in the OF-induced bovine laminitis. Despite the intense oxidative stress that *Nrf2* activation and upregulation of its downstream gene products were supposed to induce, the OF-treated group showed a gradual decrease in nuclear *Nrf2* material, indicating a decrease in the activation of this transcriptional factor in laminar tissue. This was achieved through major downregulation of *Nrf2* target gene products, including *Ho1* and *Nqo1*. This phenomenon indicates that the tendency of lame cows to climb compromises the biological response to prevailing oxidative stress and its detrimental effects on the residual laminar tissue. A sharp increase in *Keap1* abundance in the laminar tissue of OF-treated cows compared with the control cows was accompanied by a paradoxical decrease in *Nrf2* activation in the face of severe oxidative stress. A cysteine-rich protein, *Keap1*, that functions as a redox indicator, an inhibitor of *Nrf2* nuclear translocation, and a facilitator of its proteasomal degradation [43]. The apparent absence of *Nrf2* activation can therefore be due, in part, to increased *Keap1* abundance, given the predominant oxidative stress in the laminar tissue reported in this study. This is consistent with prior research indicating that the severity of oxidative stress and inflammation in *Nrf2* Knockout mice, and the exacerbated tissue injury, were amplified by *Nrf2* genetic disruption [44,45]. The resultant *Nrf2* deficiency in the current study can also be linked to laminar tissue damage in this model.

According to published studies, scientists typically concur that most cases of dairy cow laminitis arise from sub-acute rumen acidosis caused by the consumption of high-energy diets. Abaker et al. [46] reported that high-grain diet-fed SARA-induced cows show enhanced oxidative stress, as well as alterations in oxidative stress indicators in the liver and plasma, and hepatic *Nrf2* mRNA expression with increasing LPS transplanted into the bloodstream. When oxidative stress exceeds a threshold, an antioxidative response increases hepatic *Nrf2* mRNA expression [47]. In addition, *Nrf2* gene expression in liver collected from SARA-induced cows tended to decrease with increasing blood MDA [46]. Studies reported that increased oxidative stress (OS) leads to up- or downregulation of hepatic genes induced by OS [46,47]. The current results are in agreement with proteomic studies by Dong et al. [8], who have determined the presence of various oxidative stress-related proteins expressed differentially in the plasma of laminitic cows. Moreover, Zhao et al. [26] reported higher MDA levels in plasma and lower antioxidant enzyme activities in lame dairy cows, which are in agreement with our findings of systemic oxidative stress. Similar findings were reported by Li et al. [27], who observed a rise in ROS production in blood neutrophils during OF-induced laminitis, providing cellular evidence of an oxidative burst in this model. In our work, *Nrf2* also significantly decreases at both mRNA and protein levels as blood MDA increases and decreases in antioxidant enzyme activities [25]. In contrast, *Keap1* increases dramatically in the OF group than the control group, providing a mechanistic link between systemic oxidative stress markers and their underlying regulation.

This study demonstrated the ultrastructural characteristics of laminar tissue in laminitis dairy cows, indicating that adhesion between laminar epidermal basal cells (EBCs) and the basement membranes (BMs) depends on the stability of many anchoring plaques called hemidesmosomes (HDs) [48]. On an ultrastructural level, HDs are distinguished by electron-dense cytoplasmic plaques that connect epithelial cells to the basement membrane zone (BMZ) while also acting as signal transducers by connecting intermediate filaments on the plasma membrane’s cytoplasmic side with anchoring fibrils on its extracellular side [49]. Loss or damage to HDs has been documented in human bullous pemphigoid illness [50] and equine laminitis [51], resulting in improper cell–BM attachment. This segregation is the hallmark of structural alterations in dairy cows with laminitis. In their extensive review of bovine laminitis, Boosman et al. [5] found vascular changes and thrombosis to be among the pathogenic factors, which could help explain the metabolic imbalances underlying hemidesmosome loss in this study. Thus, it is suspected that the shift in the number of HDs may entail detachment of the basal epithelial cells (epidermal hoof) of laminitic dairy cows from the basement membrane and its deeper part (the dermis), which ultimately leads to the development of laminitis.

In the current study, the quantity of HDs in the laminar tissue of the OF group decreased, whereas the distance from the basal cell membrane to the central part of the hoof compact layer increased. Similar findings have been described in the horse’s laminitis [52]. Similarly, research discovered that the appearance of diseased horseshoe leaf tissue under the transmission electron microscope was similar to that of the scoring system established by [51] for the evaluation of laminitis tissue damage; that is, for both EBCs and basal BM separation, the number of missing HDs on the basement membrane is positively correlated with the degree of laminitis tissue damage. It was determined to have a positive correlation with the line scores, indicating that the ultrastructural HDs loss is linked with the animal laminitis [51].

Several mechanisms may link the observed oxidative stress and hemidesmosome loss. It is worth noting that matrix metalloproteinases (*MMPs*) were not measured in the current study, but the literature provides valuable background. Scientists currently believe that hoof separation occurs via two distinct mechanisms. In one case, glucose deprivation facilitated HDs termination, resulting in the breakdown of the epidermal basal cell cytoskeleton; in the second case, chemical activation of the epidermal basal cell–matrix metalloproteinases (*MMPs*) destroyed anchoring filaments, leaving HDs in the basal cell plasmalemma intact [53,54]. Although the precise mechanisms underlying changes in HDs on the basement membrane and glucose deprivation remain unknown, the existing literature provides some useful inspiration. In the laminitis model induced by OF, for example, the animals will exhibit obvious symptoms before lameness, including marked hyperglycemia and hypercortisolemia [55]. Scholars believe that the hooves are naturally distributed with a dense network of blood vessels that provide energy to the hooves. The laminar tissue, which is highly organized and situated at the end of circulation, has been reported to require higher glucose levels to maintain structural integrity; alternatively, glucose deficiency results in rapid HDs disassembly [53,54]. It has previously been demonstrated that glucose is required for the maintenance of laminar tissue integrity. OF dosing can induce considerable hyperglycemia, particularly at high doses, and this effect does not appear to correlate with changes in insulin quantity. The hyperglycemia caused by OF overload may indicate an unexpected failure of peripheral glucose uptake. Lamellae that are abruptly unable to transport glucose may experience HDs disintegration, leading to a laminar dermo–epidermal separation, the lesion that characterizes laminitis [56].

The current research showed that the administration of OF was correlated with shrinkage and loss of HDs in the laminar tissue of dairy cows and with the rupture of anchoring fibers. Similar ultrastructural damage can occur in laminar tissue when matrix metalloproteinases are activated. Thus, it appears that the pathophysiology of OF-induced laminitis may involve one or two processes: the activation of the constituent MMPs and the glucose scarcity in secondary epidermal lamella (SEL). Indirect data suggest that the molecular structure of BMs and anchoring fibrils is an *MMP* substrate [57], and elevated *MMP* levels have been documented in laminitic horses [54,58,59,60]. Additionally, within our research group, Ding et al. also discovered that *MMP-2* and *MMP-9* gene expression was enhanced in the same OF-induced bovine laminitis model [61]. However, the findings at that time were obtained in another study, which supports the current work by indicating that *MMP* might be associated with oxidative stress dysregulation and hemidesmosome destruction. These data suggest that the metalloproteinase family may play a role in the separation and degradation of the basement membrane in laminitic dairy cows. Overall, these observations suggest a multifactorial process in which oxidative stress, metabolic disturbance, and proteolytic enzyme activation may cause laminar failure.

During standing and walking, the cow hoof tissue is subjected to high loads, causing a significant mechanical stress at the dermal–epidermal interface. Histological changes in a laminitis cow, such as the separation of characteristic epidermal basal cells from the basement membrane, combined with ultrastructural changes, such as the loss of HDs following basement membrane damage, suggest that HDs play an important role in epidermis-dermis adhesion. Injuries to hemidesmosomes and anchoring filament weaken hoof tissue. The mechanical force of load-bearing and mobility acts on the third phalanx, leading it to spin and sink, irritating the sole dermis, and resulting in severe pain and lameness in cows [62]. In summary, dairy cows with OF-induced laminitis had reduced hemidesmosome numbers, suggesting that this may be an important factor in separating lamina densa from the epidermal basal cell membrane.

This study has several potential implications for clinical practice and therapeutic development. First, the dysregulation of the *Keap1-Nrf2* pathway suggests that pharmacological activation of *Nrf2* could represent a therapeutic strategy. *Nrf2* activators, such as sulforaphane and dimethyl fumarate, have been shown efficacy in various inflammatory and oxidative stress-mediated conditions [63,64] and thus warrant investigation in laminitis models. The reduction in *Ho1* and *Nqo1* expression indicates depletion of downstream antioxidant enzymes, suggesting that antioxidant supplementation (e.g., vitamin E or selenium) might support cellular defense mechanisms, as previously reported [25]. Additionally, rapid hemidesmosome loss highlights the need for early intervention. Practical applications may include assessing oxidative stress markers in high-risk cows, implementing dietary strategies to support antioxidant capacity, and improving management practices to minimize ruminal acidosis. However, these approaches require further investigation through controlled intervention trials before clinical implementation.

While the present study provides novel insights into oxidative stress-associated laminar damage in OF-induced dairy cow laminitis, several limitations must be acknowledged. First, the OF-induced model causes acute laminitis over 72 h, whereas naturally occurring laminitis typically develops subacutely or chronically. Second, the sample size (*n* = 6 per group), while consistent with previous studies using this model [11,12,13], is sufficient to detect significant alterations, but may not reveal more subtle molecular changes or individual variation. Third, the single 72 h time point reveals peak acute pathology but does not inform about the time course of molecular changes or potential for reversibility. Fourth, direct quantification of ROS within laminar tissue was not performed. Fifth, effect sizes and confidence intervals were not calculated, limiting the biological relevance assessment. Sixth, only selected oxidative stress-related genes and proteins were examined. These limitations indicate directions for future research: (1) longitudinal studies of *Keap1-Nrf2* dysregulation; (2) interventions with *Nrf2* activators or antioxidants; (3) functional modulation studies; (4) validation in natural laminitis cases; (5) inclusion of effect sizes and confidence intervals; (6) direct ROS quantification; and (7) minimally invasive biomarkers for clinical monitoring. Given these limitations, dysregulation of the *Keap1-Nrf2* pathway is associated with laminar injury in this experimental model rather than a definitive cause and may reflect a broader oxidative and inflammatory response.

## 5. Conclusions

**Figure 6:**
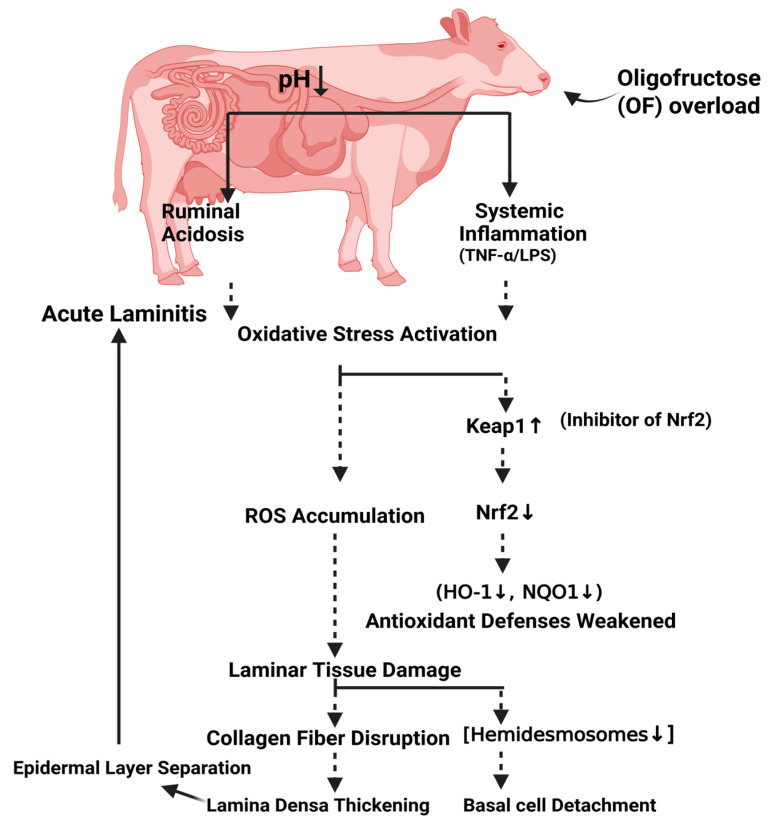
Schematic diagram illustrating the proposed oxidative stress pathway in laminar tissue during OF-induced acute laminitis in dairy cows. Following OF-overload, rumen fermentation changes, leading to rumen acidosis and systemic inflammation. Consequently, the pathway begins with the activation of the oxidative stress response and the accumulation of ROS, a key driver of acute laminitis. Elevated ROS levels increase the expression of *Keap1*, which sequesters and promotes the degradation of the master antioxidant regulator *Nrf2*. With *Nrf2* activity suppressed, the expression of cytoprotective antioxidant genes is diminished, disabling the tissue’s intrinsic defense mechanisms. This weakened cellular protection leads to laminar tissue damage, characterized by collagen fiber disruption (including reduced hemidesmosomes), separation of epidermal layers, thickening of the lamina densa, and eventual detachment of basal cells from the basement membrane. This cascade elucidates the molecular link between systemic oxidative insult and the structural failure characteristic of laminitis. The figure was created with BioRender.com.

In conclusion, this study indicates that oligofructose-induced acute laminitis in dairy cows is associated with an unbalanced oxidative stress response at both gene and protein levels in laminar tissue, accompanied by ultrastructural damage, including shrinkage and loss of hemidesmosomes and rupture of anchoring fibers (Figure 6). Together with our previous findings of increased MDA and decreased SOD, CAT, and GSH activities in the same model [25], these results provide mechanistic insight into oxidative stress in bovine laminitis, linking systemic marker changes to their underlying molecular regulation. These findings show an association between oxidative stress dysregulation and laminar injury, suggesting a broader inflammatory response during acute laminitis. Therefore, this study recommends: (1) regularly monitoring oxidative stress markers in high-risk cows; (2) supplementing antioxidants such as vitamin E and selenium; and (3) implementing dietary strategies to minimize ruminal acidosis to mitigate laminitis risk. Future research should explore intervention studies with *Nrf2* activators, validate findings in naturally occurring cases, and develop minimally invasive biomarkers. Overall, this work provides a foundation for improving prevention and control measures for bovine laminitis.

## Figures and Tables

**Table 1:** Primer sequences.

Genes	RefSeq Accession No.	Primer Sequences (5′–3′)
*Keap1*	NM_001101142.1	Forward: GGGCTACGACGGTCACACATTC Reverse: ATTCGGGTCACCTCGCTCCAG
*Nrf2*	NM_001011678.2	Forward: ACCACCCTGAAAGCACAACAGC Reverse: GAGTGGTCTGGTGATGCCATGC
*Nqo1*	NM_001034535.1	Forward: AGCGGCTCCATGTACTCTCTGC Reverse: TCCTCGGGAGTGTGCCCAATG
*Ho1*	NM_001014912.1	Forward: GCAGGCACCAGAGCTTCACAG Reverse: GAGGACCCATCGCAGGAGAGG
*GAPDH*	DQ402990	Forward: GGGTCATAAGTCCCTCCACGA Reverse: GGTCATAAGTCCCTCCACGA

**Table 2:** Mean number/μm of hemidesmosomes on epidermal basal cells (EBC) basement membrane (BM) and mean distance from the EBC plasmalemma to the center of the lamina densa in laminitic dairy cows (x ± SD).

Group	Number of Hemidesmosomes per μm on the Basal Cell Membrane (*n* = 100 μm, Counts)	Distance from the Basal Cell Membrane of the Epidermis to the Center of the Laminar Dense Zone (*n* = 100, μm)
Control	3.831 ± 1.189	0.068 ± 0.022
OF-treated	1.532 ± 1.066 **	0.098 ± 0.029 **

** indicated *p* < 0.01, an extremely significant difference.

## Data Availability

All data generated or analyzed during the study are included in this published article.

## Supplementary Materials

The following supporting information can be downloaded at: https://www.mdpi.com/article/10.3390/ani16060980/s1, Figure S1: Keap1, Figure S2: Nrf2, Figure S3: Nqo1, Figure S4: Ho1.

## Abbreviations

ARE	Antioxidant Response Element
BM	Basement Membrane
CAT	Catalase
cDNA	Complementary DNA
DEPC	Diethyl Pyrocarbonate
ECM	Extracellular Matrix
GAPDH	Glyceraldehyde-3-Phosphate Dehydrogenase
GSH	Glutathione
HDs	Hemidesmosomes
Ho1	Heme Oxygenase-1
Keap1	Kelch-like ECH-Associated Protein 1
MDA	Malondialdehyde
MMPs	Matrix Metalloproteinases
Nqo1	NAD(P)H:Quinone Oxidoreductase 1
Nrf2	Nuclear Factor Erythroid 2-Related Factor 2
OF	Oligofructose
P3	Third Phalanx

## References

[1] Tuniyazi M., Tang R., Hu X., Zhang N. (2024). Methylated tirilazad may mitigate oligofructose-induced laminitis in horses. Front. Microbiol..

[2] Passos L.T., Bettencourt A.F., Ritt L.A., Canozzi M.E.A., Fischer V. (2023). Systematic review of the relationship between rumen acidosis and laminitis in cattle. Res. Vet. Sci..

[3] Serİn H., Körez M.K. (2024). Laminitis in cattle: A bibliometric analysis. Res. Pract. Vet. Anim. Sci..

[4] Bojkovski J., Nedić S., Arsić S., Vujanac I., Prodanović R., Mitrović A., Đurić M., Bugarski D., Panousis N.K., Kalaitzakis E. (2023). Pathogenesis of laminitis in dairy cows. Vet. Žur. Republik. Srpsk..

[5] Boosman R., Nemeth F., Gruys E. (1991). Bovine laminitis: Clinical aspects, pathology and pathogenesis with reference to acute equine laminitis. Vet. Q..

[6] Bäßler S.C., Kenéz Á., Scheu T., Koch C., Meyer U., Dänicke S., Huber K. (2021). Association between alterations in plasma metabolome profiles and laminitis in intensively finished Holstein bulls in a randomized controlled study. Sci. Rep..

[7] Danscher A.M., Toelboell T.H., Wattle O. (2010). Biomechanics and histology of bovine claw suspensory tissue in early acute laminitis. J. Dairy Sci..

[8] Dong S.W., Zhang S.D., Wang D.S., Wang H., Shang X.F., Yan P., Yan Z.T., Yang Z.Q. (2015). Comparative proteomics analysis provide novel insight into laminitis in Chinese Holstein cows. BMC Vet. Res..

[9] Randall L.V., Green M.J., Huxley J.N. (2018). Use of statistical modelling to investigate the pathogenesis of claw horn disruption lesions in dairy cattle. Vet. J..

[10] Greenough P.R. (2007). Bovine Laminitis and Lameness: A Hands on Approach.

[11] Thoefner M.B., Pollitt C.C., Van-Eps A.W., Milinovich G.J., Trott D.J., Wattle O., Andersen P.H. (2004). Acute bovine laminitis: A new induction model using alimentary oligofructose overload. J. Dairy Sci..

[12] Danscher A.M., Enemark J.M.D., Telezhenko E., Capion N., Ekstrom C.T., Thoefner M.B. (2009). Oligofructose overload induces lameness in cattle. J. Dairy Sci..

[13] Thoefner M.B., Wattle O., Pollitt C.C., French K.R., Nielsen S.S. (2005). Histopathology of oligofructose-induced acute laminitis in heifers. J. Dairy Sci..

[14] Mendes H.M., Casagrande F.P., Lima I.R., Souza C.H., Gontijo L.D., Alves G.E., Vasconcelos A.C., Faleiros R.R. (2013). Histopathology of dairy cows’ hooves with signs of naturally acquired laminitis. Pesqui. Vet. Bras..

[15] Leise B.S., Faleiros R.R., Watts M., Johnson P.J., Black S.J., Belknap J.K. (2011). Laminar inflammatory gene expression in the carbohydrate overload model of equine laminitis. Equine Vet. J..

[16] Dern K., Van Eps A., Wittum T., Watts M., Pollitt C., Belknap J. (2018). Effect of continuous digital hypothermia on lamellar inflammatory signaling when applied at a clinically-relevant timepoint in the oligofructose laminitis model. J. Vet. Int. Med..

[17] Raber M., Lischer C.J., Geyer H., Ossent P. (2004). The bovine digital cushion—A descriptive anatomical study. Vet. J..

[18] Vermunt J. (1992). “Subclinical” laminitis in dairy cattle. N. Z. Vet. J..

[19] Zamith Cunha R., Gobbo F., Morini M., Zannoni A., Mainardi C., D’arpe L., Gramenzi A., Chiocchetti R. (2025). Distribution of endocannabinoid system receptors in the equine hoof: Dysregulation as a potential therapeutic target for laminitis. Histochem. Cell Bio..

[20] Hayat M.A., Ding J., Zhang X., Liu T., Zhang J., Wang H.B. (2025). Enhanced apoptosis in damaged laminar tissue of acute laminitis induced by oligofructose overload in dairy cows. Vet. Immunol. Immunopathol..

[21] Caliri A.W., Tommasi S., Besaratinia A. (2021). Relationships among smoking, oxidative stress, inflammation, macromolecular damage, and cancer. Mutat. Res./Rev. Mutat. Res..

[22] Kıran T.R., Otlu O., Karabulut A.B. (2023). Oxidative stress and antioxidants in health and disease. J. Lab. Med..

[23] Devecİ M., Erdal H. (2022). Determination of dynamic thiol-disulfide levels in dairy cattle with foot disease Dinamičke razine tiol-disulfida u mliječnih goveda s bolestima papaka. Vet. Arhiv..

[24] Heinecke L.F., Grzanna M.W., Au A.Y., Mochal C.A., Rashmir-Raven A., Frondoza C.G. (2010). Inhibition of cyclooxygenase-2 expression and prostaglandin E2 production in chondrocytes by avocado soybean unsaponifiables and epigallocatechin gallate. Osteoarthritis Cartilage..

[25] Hayat M.A., Ding J., Li Y.U., Zhang X., Zhang J.I., Li S., Wang H.B. (2020). Determination of the activity of selected antioxidant enzymes during bovine laminitis, induced by oligofructose overload. Med. Weter..

[26] Zhao X.J., Wang X.Y., Wang J.H., Wang Z.Y., Wang L., Wang Z.H. (2015). Oxidative stress and imbalance of mineral metabolism contribute to lameness in dairy cows. Bio. Trace Elem. Res..

[27] Li S., Ding J., Jiang L., Hayat M.A., Song Q., Li Y., Zhang X., Zhang J. (2020). Dynamic ROS production and gene expression of heifers blood neutrophil in a oligofructose overload model. Front. Vet. Sci..

[28] Hong Y., Boiti A., Vallone D., Foulkes N.S. (2024). Reactive oxygen species signaling and oxidative stress: Transcriptional regulation and evolution. Antioxidants.

[29] Liu S., Pi J., Zhang Q. (2022). Signal amplification in the KEAP1-NRF2-ARE antioxidant response pathway. Redox Bio..

[30] Cui L., Duan J., Mao P., Zhong J., He S., Dong J., Liu K., Guo L., Li J., Wang H. (2025). Meloxicam alleviates oxidative stress through Nrf2/HO-1 activation in bovine endometrial epithelial cells. Vet. Sci..

[31] Annie-Mathew A.S., Prem-Santhosh S., Jayasuriya R., Ganesh G., Ramkumar K.M., Sarada D.V.L. (2021). The pivotal role of Nrf2 activators in adipocyte biology. Pharmacol. Res..

[32] Khan M.Z., Li L., Zhan Y., Binjiang H., Liu X., Kou X., Khan A., Qadeer A., Ullah Q., Alzahrani K.J. (2025). Targeting Nrf2/KEAP1 signaling pathway using bioactive compounds to combat mastitis. Front. Immunol..

[33] Yin C., Pettigrew A., Loftus J.P., Black S.J., Belknap J.K. (2009). Tissue concentrations of 4-HNE in the black walnut extract model of laminitis: Indication of oxidant stress in affected laminae. Vet. Immunol. Immunopathol..

[34] Loftus J.P., Belknap J.K., Stankiewicz K.M., Black S.J. (2007). Laminar xanthine oxidase, superoxide dismutase and catalase activities in the prodromal stage of black-walnut induced equine laminitis. Equine Vet. J..

[35] Sprecher D.E.A., Hostetler D.E., Kaneene J.B. (1997). A lameness scoring system that uses posture and gait to predict dairy cattle reproductive performance. Theriogenol.

[36] Edmonson A.J., Lean I.J., Weaver L.D., Farver T., Webster G. (1989). A body condition scoring chart for Holstein dairy cows. J. Dairy Sci..

[37] Ding J., Li S., Jiang L., Li Y., Zhang X., Song Q., Hayat M.A., Zhang J.T., Wang H. (2020). Laminar inflammation responses in the oligofructose overload induced model of bovine laminitis. Front. Vet. Sci..

[38] Hayat M.A., Ding J., Zhang X., Liu T., Zhang J., Bokhari S.G., Akbar H., Wang H. (2023). Enhanced Autophagy in Damaged Laminar Tissue of Acute Laminitis Induced by Oligofructose Overloading in Dairy Cows. Animals.

[39] Kim M.J., Jeon J.H. (2022). Recent advances in understanding Nrf2 agonism and its potential clinical application to metabolic and inflammatory diseases. Int. J. Mol. Sci..

[40] Sharma V., Mehdi M.M. (2023). Oxidative stress, inflammation and hormesis: The role of dietary and lifestyle modifications on aging. Neurochem. Int..

[41] Tkaczenko H., Kurhaluk N. (2025). Antioxidant-rich functional foods and exercise: Unlocking metabolic health through Nrf2 and related pathways. Int. J. Mol. Sci..

[42] Zhang M., Wang J., Liu R., Wang Q., Qin S., Chen Y., Li W. (2024). The role of Keap1-Nrf2 signaling pathway in the treatment of respiratory diseases and the research progress on targeted drugs. Heliyon.

[43] Crisman E., Duarte P., Dauden E., Cuadrado A., Rodríguez-Franco M.I., López M.G., León R. (2023). KEAP1-NRF2 protein–protein interaction inhibitors: Design, pharmacological properties and therapeutic potential. Med. Res. Rev..

[44] Yoh K., Hirayama A., Ishizaki K., Yamada A., Takeuchi M., Yamagishi S.I., Morito N., Nakano T., Ojima M., Shimohata H. (2008). Hyperglycemia induces oxidative and nitrosative stress and increases renal functional impairment in Nrf2-deficient mice. Genes Cells.

[45] Yoh K., Itoh K., Enomoto A., Hirayama A., Yamaguchi N., Kobayashi M., Morito N., Koyama A., Yamamoto M., Takahashi S. (2001). Nrf2-deficient female mice develop lupus-like autoimmune nephritis. Kidney Int..

[46] Abaker J.A., Xu T.L., Jin D., Chang G.J., Zhang K., Shen X.Z. (2017). Lipopolysaccharide derived from the digestive tract provokes oxidative stress in the liver of dairy cows fed a high-grain diet. J. Dairy Sci..

[47] Gessner D.K., Schlegel G., Keller J., Schwarz F.J., Ringseis R., Eder K. (2013). Expression of target genes of nuclear factor E2-related factor 2 in the liver of dairy cows in the transition period and at different stages of lactation. J. Dairy Sci..

[48] Elliott J., Bailey S.R. (2023). A review of cellular and molecular mechanisms in endocrinopathic, sepsis-related and supporting limb equine laminitis. Equine Vet. J..

[49] Borradori L., Sonnenberg A. (1996). Hemidesmosomes: Roles in adhesion, signaling and human diseases. Curr. Opin. Cell Bio..

[50] Lin M.S., Mascaó J.M., Liu Z., Espana A., Diaz L.A. (1997). The desmosome and hemidesmosome in cutaneous autoimmunity. Clin. Exp. Immunol..

[51] French K.R., Pollitt C.C. (2004). Equine laminitis: Loss of hemidesmosomes in hoof secondary epidermal lamellae correlates to dose in an oligofructose induction model: An ultrastructural study. Equine Vet. J..

[52] Wang L., Pawlak E.A., Johnson P.J., Belknap J.K., Eades S., Stack S., Cousin H., Black S.J. (2013). Impact of laminitis on the canonical Wnt signaling pathway in basal epithelial cells of the equine digital laminae. PLoS ONE.

[53] French K.R., Pollitt C.C. (2004). Equine laminitis: Glucose deprivation and MMP activation induce dermo-epidermal separation in vitro. Equine Vet. J..

[54] Kyaw-Tanner M., Pollitt C.C. (2004). Equine laminitis: Increased transcription of matrix metalloproteinase-2 (MMP-2) occurs during the developmental phase. Equine Vet. J..

[55] Johnson P.J. (2017). Endocrine and metabolic dysregulation in laminitis: Role of corticosteroids. Equine Laminitis.

[56] Pass M.A., Pollitt S., Pollitt C.C. (1998). Decreased glucose metabolism causes separation of hoof lamellae in vitro: A trigger for laminitis?. Equine Vet. J..

[57] Giannelli G., Falk-Marzillier J., Schiraldi O., Stetler-Stevenson W.G., Quaranta V. (1997). Induction of cell migration by matrix metalloprotease-2 cleavage of laminin-5. Science.

[58] Wang L., Pawlak E.A., Johnson P.J., Belknap J.K., Alfandari D., Black S.J. (2014). Expression and activity of collagenases in the digital laminae of horses with carbohydrate overload-induced acute laminitis. J. Vet. Int. Med..

[59] Loftus J.P., Johnson P.J., Belknap J.K., Pettigrew A., Black S.J. (2009). Leukocyte-derived and endogenous matrix metalloproteinases in the lamellae of horses with naturally acquired and experimentally induced laminitis. Vet. Immunol. Immunopathol..

[60] Clutterbuck A.L., Harris P., Allaway D., Mobasheri A. (2010). Matrix metalloproteinases in inflammatory pathologies of the horse. Vet. J..

[61] Ding J., Shi M., Wang L., Qi D., Tao Z., Hayat M.A., Liu T., Zhang J.T., Wang H. (2020). Gene expression of metalloproteinases and endogenous inhibitors in the lamellae of dairy heifers with oligofructose-induced laminitis. Front. Vet. Sci..

[62] Laat M.A.D., Pollitt C.C. (2019). Ultrastructural examination of basement membrane pathology in horses with insulin-induced laminitis. Domest. Anim. Endocrinol..

[63] Khan M.Z., Li S., Ullah A., Li Y., Abohashrh M., Alzahrani F.M., Alzahrani K.J., Alsharif K.F., Wang C., Ma Q. (2025). Therapeutic Agents Targeting the Nrf2 Signaling Pathway to Combat Oxidative Stress and Intestinal Inflammation in Veterinary and Translational Medicine. Vet. Sci..

[64] Thiruvengadam M., Venkidasamy B., Subramanian U., Samynathan R., Ali Shariati M., Rebezov M., Girish S., Thangavel S., Dhanapal A.R., Fedoseeva N. (2021). Bioactive compounds in oxidative stress-mediated diseases: Targeting the NRF2/ARE signaling pathway and epigenetic regulation. Antioxidant.

