# Supplementary material for: Oxidative Stress and Ultrastructural Changes in Laminar Tissue of Dairy Cows with Acute Laminitis Induced by Oligofructose Overload
Source: Animals (Basel). 2026 Mar 20;16(6):980. doi: 10.3390/ani16060980 (PMC13023642; doi:10.3390/ani16060980)

Supplementary Materials Figures (S1-S4) & claudication score

Western blots Images Data (Keap1, Nrf2, Nqo1, and Ho1)

Figure S1:keap1

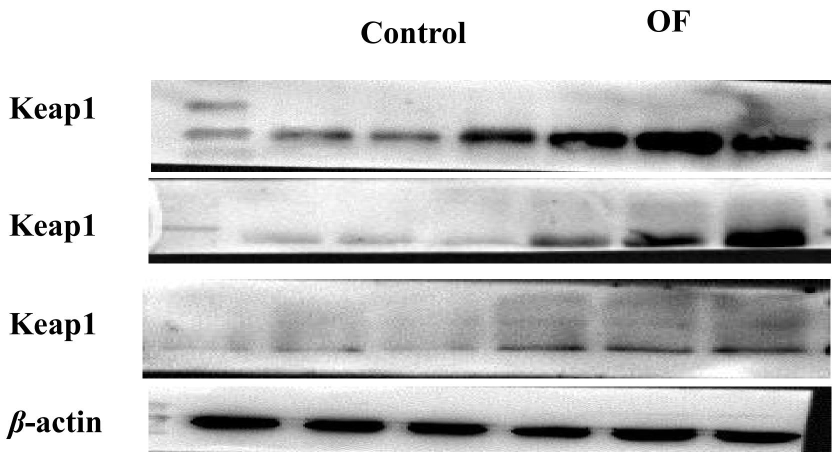

Figure S2:Nrf2

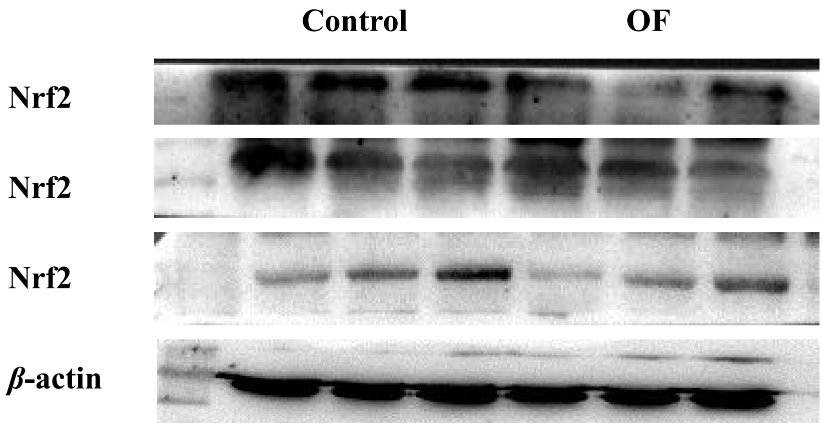

<u>Figure S3:Nqo1</u>

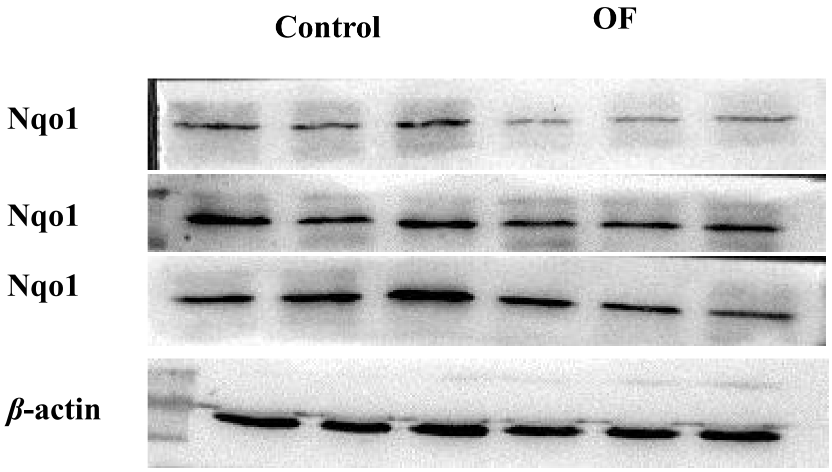

<u>Figure S4:Ho1</u>

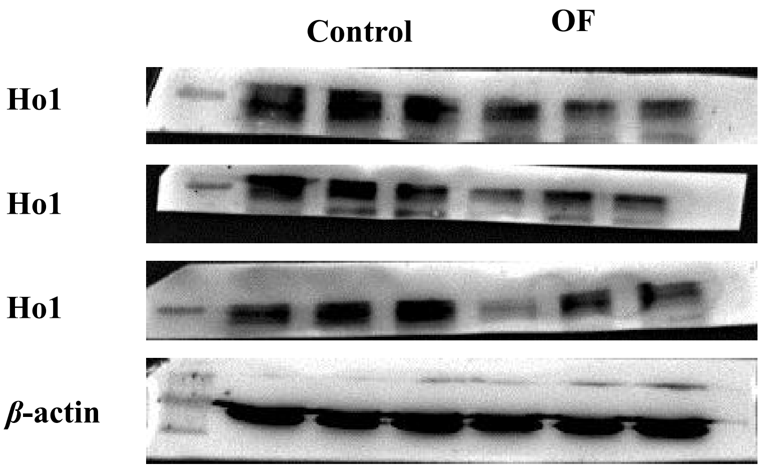

Supplement: Supplementary file 1 [file animals-16-00980-s001.zip › animals-4155045-supplementary.pdf]
